# Fermented Yeast Complex Extract Promotes Hair Regrowth by Decreasing Oxidative Stress

**DOI:** 10.3390/antiox14121503

**Published:** 2025-12-14

**Authors:** Kyung-A Byun, Chang Hu Choi, Seyeon Oh, Jimin Hyun, Kuk Hui Son, Kyunghee Byun

**Affiliations:** 1 Department of Anatomy & Cell Biology, College of Medicine, Gachon University, Incheon 21936, Republic of Korea; 2 LIBON Inc., Incheon 22006, Republic of Korea; 3 Department of Thoracic and Cardiovascular Surgery, Gachon University Gil Medical Center, Gachon University, Incheon 21565, Republic of Korea; 4 Functional Cellular Networks Laboratory, Lee Gil Ya Cancer and Diabetes Institute, Gachon University, Incheon 21999, Republic of Korea; 5 Major of Food Science and Nutrition, Pukyong National University, Busan 48513, Republic of Korea; 6 Department of Health Sciences and Technology, Gachon Advanced Institute for Health & Sciences and Technology (GAIHST), Gachon University, Incheon 21999, Republic of Korea

**Keywords:** fermented yeast complex extract, hair regrowth, oxidative stress

## Abstract

Hair growth is orchestrated by a complex cycle comprising the anagen, catagen, telogen, and exogen phases that are largely regulated by dermal papilla cells (DPCs). The disruption of oxidative balance and inflammation impairs follicle function and regeneration. Fermented yeast complex extract (FYCE) is a bioactive material derived from enzymatically hydrolyzed yeast and collagen substrates through a two-step fermentation with *Lactobacillus brevis* and *Lactobacillus plantarum*, enriched in antioxidant amino acids such as γ-aminobutyric acid (GABA) and L-alanine. In this study, we evaluated the effect of FYCE on hair regrowth, with a focus on its modulation of oxidative stress and inflammatory pathways in hydrogen peroxide (H_2_O_2_)-treated DPCs. FYCE treatment significantly enhanced NRF2 expression (3.2-fold compared to H_2_O_2_-treated DPCs), a central transcription factor controlling antioxidant defense, and concomitantly suppressed NF-κB activity (0.6-fold compared to H_2_O_2_-treated DPCs), a key mediator of inflammation. Importantly, FYCE also attenuated the activation of the NLRP3 inflammasome, as evidenced by the decreased expression levels of its molecular components. Complementary studies showed that FYCE increased IGF-1 (5.4-fold compared to H_2_O_2_-treated DPCs), Wnt10b (1.8-fold compared to H_2_O_2_-treated DPCs), and Wnt3a (2.9-fold compared to H_2_O_2_-treated DPCs), and stabilized β-catenin (2.8-fold compared to H_2_O_2_-treated DPCs). FYCE also showed these changes in the shaved animal skin, which was associated with increased hair follicle number (1.6-fold compared to the water-administered control group) and the anagen phase (3.0-fold compared to the water-administered control group). Collectively, our results suggest that FYCE promotes hair regrowth through the dual modulation of antioxidative and anti-inflammatory pathways, specifically by activating NRF2, inhibiting NF-κB signaling, and downregulating the NLRP3 inflammasome. These findings support FYCE as a promising candidate for further investigation as a treatment to prevent or reverse hair loss, with in vivo and clinical studies substantiating its efficacy and safety.

## 1. Introduction

Hair growth is a continuous process consisting of four distinct phases: anagen (growth), catagen (regression), telogen (rest), and exogen (shedding) [1]. Dermal papilla cells (DPCs), located at the base of the hair follicle (HF), control the hair growth cycle by engaging in essential reciprocal communication with hair follicle stem cells (HFSCs) and other epithelial components [2].

The cyclical activity of HFs is critically dependent on paracrine signaling pathways, most notably the Wnt/β-catenin pathway, which is pivotal for follicle morphogenesis and the induction of a new growth phase [3,4]. By activating quiescent HFSCs and stimulating epithelial cell proliferation, DPCs drive the transition from the resting telogen to the active anagen phase [5]. DPCs secrete various growth factors, such as insulin-like growth factor 1 (IGF-1), which increase the proliferation of hair follicular cells [6,7].

Increased oxidative stress results in hair loss [8]. Oxidative stress in the skin increases due to external factors such as ultraviolet radiation and intrinsic factors such as chronic inflammation and aging [9,10]. Oxidative stress induces the abnormal morphogenesis of hair follicles and hair cycle dysregulation [9]. IGF-1 is decreased by inflammatory cytokines, such as tumor necrosis factor-alpha (TNF-α) and interleukin-6 (IL-6), and increased oxidative stress [11]. Hydrogen peroxide (H_2_O_2_)-induced oxidative stress decreases IGF-1 levels in DPCs [12].

During the catagen phase, increased ROS levels lead to hair follicle degeneration [13]. ROS levels are higher in bald scalps than in non-bald scalps [14,15].

Nuclear factor erythroid 2-related factor 2 (NRF2) is essential for decreasing oxidative stress through the upregulation of various antioxidant enzymes [16]. Increased oxidative stress results in increased nuclear factor-κB (NF-κB) activity [17]. NF-κB is a key transcription factor to upregulate inflammatory cytokines such as TNF-α, IL-1β, and IL-6 [18].

NF-κB also promotes inflammasome formation. The most well-known inflammasome, the NOD-like receptor protein 3 (NLRP3) inflammasome, consists of NLRP3, an apoptosis-associated speck-like protein containing CARD (ASC), and pro-caspase 1 [19]. Through NLRP3 inflammasome formation, pro-caspase 1 is activated to the mature form of caspase 1, which converts pro-IL-1β to IL-1β [20].

Gamma-aminobutyric acid (GABA) decreases oxidative stress and NF-κB in H_2_O_2_-treated human umbilical vein endothelial cells [21]. GABA also decreases oxidative stress by upregulating NRF2 [22,23].

L-alanine also shows an antioxidant effect, which leads to decreased H_2_O_2_-induced cell toxicity via upregulation of heme oxygenase-1 [24]. L-alanine supplementation also decreases TNF-α and NF-κB activation [25].

Fermented yeast complex extract (FYCE) is a novel bioactive material obtained through a two-step lactic acid bacterial fermentation of enzymatically hydrolyzed yeast and collagen substrates. Amino acid analysis revealed that FYCE contains high levels of GABA and L-alanine. Unlike conventional yeast extracts (YEs), which are typically produced by the single-step autolysis of Saccharomyces cerevisiae without secondary fermentation, FYCE is prepared from enzymatically hydrolyzed yeast combined with fish collagen and subsequently fermented in two stages using *Lactobacillus brevis* BJ20 and *L. plantarum* BJ21. This dual fermentation process promotes the bioconversion of glutamate into GABA and the formation of low-molecular-weight peptides derived from collagen, resulting in a composition and functionality distinct from those of standard YE. In addition, the inclusion of kelp extract provides extra bioactive peptides and trace minerals such as iodine, further enhancing the antioxidative and anti-inflammatory potential of FYCE.

Previous studies have reported the antioxidant effects of GABA and L-alanine. Increased oxidative stress has been implicated in hair follicle damage and subsequent hair loss. Based on these findings, we hypothesized that GABA- and L-alanine-containing FYCE could mitigate hair loss by preventing the decline in IGF-1 in DPCs under oxidative stress. The proposed mechanism is that GABA and L-alanine in FYCE increase the expression of NRF2, a key regulator of the antioxidant response. This enhanced NRF2 activity reduces oxidative stress, which in turn decreases the activity of NF-κB. The suppression of NF-κB leads to a reduction in the expression of pro-inflammatory cytokines, particularly TNF-α and IL-6. Furthermore, the decreased oxidative stress and subsequent reduction in NF-κB activity are expected to inhibit the formation of the NLRP3 inflammasome, thereby lowering the production of IL-1β. By suppressing this inflammatory cascade, we hypothesized that the decrease in IGF-1 levels, a known consequence of inflammation, could be reversed, thereby promoting hair growth. To test this hypothesis, we conducted experiments using H_2_O_2_-treated DPCs and a gentle anagen-induced mouse model. We treated the cells and mice with the FYCE, GABA alone, and L-alanine alone to evaluate their effects on reducing oxidative stress and increasing IGF-1 secretion, and a comparative analysis was performed among the FYCE, GABA, and L-alanine treatment groups to assess their relative efficacy.

## 2. Materials and Methods

### 2.1. FYCE Preparation and Analysis

#### 2.1.1. FYCE Preparation

FYCE was prepared by Marine Bioprocess Co., Ltd. (Busan, Republic of Korea) via a two-step fermentation process using *Lactobacillus brevis* BJ20 (accession no. KCTC 11377BP) and *Lactobacillus plantarum* BJ21 (accession no. KCTC 18911P).

Inter Yeast^®^ Vital S CW powder was obtained from Fine Bs Co., Ltd. (Seoul, Republic of Korea), and fish collagen was purchased from Geltech Co., Ltd. (Busan, Republic of Korea). Before fermentation, Inter Yeast^®^ Vital S CW powder (5%, *w*/*w*) and fish collagen (approximately 0.1 g per gram of the final total culture weight) were dissolved in distilled water. The mixture was enzymatically hydrolyzed with 0.2% (*w*/*w*) FoodPro^®^ Alkaline Protease (Bisionbiochem Co., Ltd., Seoul, Republic of Korea) at pH 8.7 and incubated at 55 ± 2 °C for 3 h with shaking at 150 rpm. The hydrolysate was used as the base of the fermentation medium and deactivated by heat during autoclaving.

The seed cultures of *L. brevis* BJ20 and *L. plantarum* BJ21 were prepared separately. Each strain was inoculated into sterilized medium containing 3% yeast extract (Choheung Co., Ltd., Ansan, Republic of Korea), 1% glucose (Choheung Co., Ltd., Ansan, Republic of Korea), 1% monosodium glutamate (CJ CheilJedang Corp., Seoul, Republic of Korea), and 95% distilled water. The medium was autoclaved at 121 °C for 15 min, inoculated with 0.02% (*v*/*v*) of each strain, and incubated at 37 °C for 24 h without shaking.

The main fermentation medium consisted of 4% glucose (*w*/*w*, Choheung Co., Ltd., Ansan, Republic of Korea), 0.5% rice bran (*w*/*w*, I-rice Co., Ltd., Hwaseong, Republic of Korea), 3% sea tangle extract (*w*/*w*), 1% L-glutamic acid (*w*/*w*, CSF inc., Busan, Republic of Korea), 2% monosodium glutamate (*w*/*w*, CJ CheilJedang Corp., Seoul, Republic of Korea), 68.5% hydrolysate (*w*/*w*) and 21% distilled water (*w*/*w*). The medium was autoclaved at 121 °C for 15 min.

For the first fermentation step, a 10% (*w*/*w*) *L. brevis* BJ20 seed culture was added to the sterilized main fermentation medium containing brewer’s yeast hydrolysate and additional nutrients. Fermentation was performed at 36.5 °C and 150 rpm for 24 h. Subsequently, 10% (*w*/*w*) of the *L. plantarum* BJ21 seed culture was added, and fermentation was continued under the same conditions for another 24 h.

The culture was filtered, heat-treated, and spray-dried to obtain FYCE powder samples. Spray drying was performed at an inlet temperature of 190 ± 30 °C and an outlet temperature of 90 ± 10 °C (Figure S1).

#### 2.1.2. FYCE Analysis

The FYCE was hydrolyzed and analyzed for amino acid composition using an amino acid analyzer (L-8900; Hitachi, Tokyo, Japan) and HPLC (Dionex UltiMate 3000 series, Thermo Fisher Scientific, Waltham, MA, USA) following acid hydrolysis. Quantification was performed using external calibration with certified GABA and alanine standards, and calibration curves were established within the validated concentration range (100–200 mg/kg). Each production batch of FYCE was standardized based on the contents of GABA and alanine, with acceptance criteria defined as GABA ≥ 50 mg/g and alanine ≥ 10 mg/g, ensuring batch-to-batch compliance within 80–120% of the labeled specification (Table S1).

Additional quality parameters, including moisture content, color, odor, and microbiological purity, were assessed to confirm product consistency and safety. Microbiological analysis confirmed the absence of coliforms, Salmonella spp., Escherichia coli, and Bacillus cereus, meeting Korean food safety standards (Table S2).

#### 2.1.3. Physicochemical Characterization of FYCE

An FYCE stock dispersion (100 mg/mL, *w*/*v*) was prepared by weighing 3 g of FYCE into a 50 mL polypropylene conical tube and adding 20 mL of distilled water (DW). The mixture was vortexed, and DW was added to adjust the final volume to 30 mL. The suspension was vortexed again to obtain a homogeneous 100 mg/mL stock formulation. This stock was diluted to 1 mg/mL before analysis.

The particle size distribution, polydispersity index (PDI), and zeta potential of the FYCE were analyzed at the Pusan National University Core Research Facility (Busan, Republic of Korea) using a Zetasizer Nano ZSP (Malvern Instruments, Worcestershire, UK). Microscale particle-size distribution was measured in the same facility using a LS 13 320 particle-size analyzer (Beckman Coulter, Brea, CA, USA). FYCE dispersions were submitted without further processing, and particle-size parameters (D10, D50, D90, mean, mode, and coefficient of variation) were obtained using the Fraunhofer optical model.

### 2.2. In Vitro Experiments

#### 2.2.1. Cell Culture

Human follicle dermal papilla cells (HFDPC; PromoCell, Heidelberg, Germany) were cultured in Follicle Dermal Papilla Cell Growth Medium (PromoCell). The cells were maintained in a humidified environment with 5% CO_2_ at 37 °C until approximately 80% confluence.

#### 2.2.2. Experimental Design for FYCE Treatment

To investigate the condition of oxidative stress, DPCs were treated with 0, 100, 150, and 200 μM of H_2_O_2_ for 24 h. The 8-OHdG levels significantly increased at concentrations above 200 μM; thus, this concentration was used in the experiment. Cells under oxidative stress were exposed to FYCE at concentrations of 0–10 mg/mL for an additional 48 h to determine cytotoxicity. The effective concentrations of FYCE were determined based on the changes in NRF2 and 8-OHdG levels at 0.2, 0.4, 0.8, and 1 mg/mL. Similar changes were observed at concentrations above 0.8 mg/mL, which was used in this experiment. GABA and L-alanine were added at 5% and 1%, respectively, to match the concentrations in FYCE.

### 2.3. Cell Viability Assessment

To evaluate the cytotoxicity of FYCE, the lyophilized FYCE powder was directly dissolved and diluted in the culture medium to obtain the final working concentrations (0–10 mg/mL). The cells exposed to oxidative stress were treated with FYCE for 48 h. After treatment, the CCK-8 reagent (TransGen Biotech Co., Ltd., Beijing, China) was added to each well, and the absorbance was measured at 450 nm after 2 h of incubation.

### 2.4. In Vivo Experiments

#### 2.4.1. Mouse Maintenance and FYCE Treatment

During the study, mice were housed in a controlled environment maintained at a constant temperature of 20–24 °C and humidity of 45–55%, with free access to standard laboratory food and water. All animal experimental protocols were approved by the Institutional Animal Care and Use Committee (IACUC) of Gachon University (Approval Number: LCDI-2025-0027). The experimental procedures were performed in accordance with the national and institutional guidelines for ethical animal experimentation.

Six-week-old male C57BL/6N mice were purchased from Orient Bio, Inc. (Seongnam, Republic of Korea). After a one-week acclimation period, all animals were shaved one day before oral administration. Shaved animals with inconsistent hair cycles were excluded from this experiment. The animals with identical hair cycles were randomly divided into six groups of five mice for oral administration [26].

Six groups were orally administered water; FYCE 300, 400, and 500 mg/kg; GABA (20 mg/kg); or L-alanine (4 mg/kg) for 21 days at a volume of 5 mL/kg. After 21 days of oral administration, the animals were photographed to identify hair growth areas, and skin samples from the same site were excised and fixed with 4% paraformaldehyde for histological analysis or flash-frozen for molecular analysis.

#### 2.4.2. Hair Growth Area

The hair growth areas were compared and analyzed by capturing their appearance using a camera. Photographs were taken at end point under fixed lighting, distance, and angle. Hair growth in the shaved dorsal region was quantitatively evaluated using ImageJ software (National Institutes of Health, Bethesda, MD, USA). The images quantified the area covered by regrowth hair relative to the total shaved area, and the hair growth area was calculated as the area filled with hair compared to the water-treated control.

### 2.5. Sample Preparation

#### 2.5.1. Protein Isolation

Proteins were extracted using EzRIPA buffer (ATTO Corporation, Tokyo, Japan), and their concentrations were quantified using a bicinchoninic acid assay kit (Thermo Fisher Scientific, Waltham, MA, USA). All procedures were performed according to the manufacturer’s instructions.

#### 2.5.2. RNA Extraction

Total RNA was extracted using the RNAiso reagent (TAKARA, Tokyo, Japan), and the RNA concentration was measured using a Nanodrop spectrophotometer (Thermo Fisher Scientific).

#### 2.5.3. Paraffin-Embedded Skin Tissue Blocks

Tissue samples were fixed in 4% paraformaldehyde (Sigma-Aldrich, St. Louis, MO, USA) for 72 h and processed using a tissue processor (Leica, Wetzlar, Germany). They were then dehydrated, embedded in paraffin blocks, and sectioned at 7 µm thickness using a microtome. The sections were mounted on slides and dried overnight at 60 °C.

### 2.6. Enzyme-Linked Immunosorbent Assay

Microplates were coated with protein antigens diluted in bicarbonate–carbonate buffer (pH 9.6, sodium counter ion) and incubated overnight at 4 °C. The following day, nonspecific binding was blocked with a 5% skim milk solution. Primary antibodies (Table S1, diluted in PBS) were then added and incubated overnight at 4 °C. After washing, horseradish peroxidase (HRP)-conjugated secondary antibodies (1:10,000; Vector Laboratories, Burlingame, CA, USA) were added and incubated for 3 h at room temperature. Color development was performed using tetramethylbenzidine solution (Sigma-Aldrich) at room temperature for 10 min, and the reaction was terminated with 1 M sulfuric acid (Sigma-Aldrich). The absorbance was measured at 450 nm using a microplate reader (Thermo Fisher Scientific, Waltham, MA, USA).

### 2.7. Quantitative Reverse-Transcription–Polymerase Chain Reaction (RT-qPCR)

Total RNA (1 μg) was reverse transcribed to synthesize complementary DNA (TAKARA) according to the manufacturer’s instructions. Quantitative reverse-transcription–polymerase chain reaction (RT-qPCR) was performed using the QuantStudio™ 3 Real-Time PCR System (Thermo Fisher Scientific) and SYBR Green Premix (TAKARA). Gene expression was analyzed using the ΔΔCt method and normalized to *ACTB* as a reference gene.

### 2.8. Western Blotting

Equal amounts of protein obtained from cell lysates or skin tissues were separated using SDS-PAGE and transferred to 0.45 µm PVDF membranes. The membrane was blocked with 5% skim milk, washed three times, and incubated overnight at 4 °C with primary antibodies (Table S1). Additional washes were followed by incubation with HRP-conjugated secondary antibodies (1:10,000; Vector Laboratories) for 1 h at room temperature. Protein expression was visualized using a chemiluminescent detection reagent, and images were acquired using a ChemiDoc imaging system (Bio-Rad, Hercules, CA, USA). Band intensities were analyzed semi-quantitatively using ImageJ version 1.53s (NIH, Bethesda, MD, USA). The signal intensities of each target protein were normalized to the ratio of the endogenous control, such as β-actin.

### 2.9. Staining

#### 2.9.1. Immunocytochemistry

DPCs were washed with PBS and incubated in a serum solution for 1 h at room temperature to block nonspecific binding. The cells were then incubated overnight at 4 °C with primary antibodies (Table S1). The following day, after washing, the cells were incubated with Alexa Fluor^®^ 488-conjugated secondary antibodies (Invitrogen, Carlsbad, CA, USA) for 1 h at room temperature. Nuclei were counterstained with DAPI (Sigma-Aldrich) for 30 s and mounted using Vectashield mounting solution (Vector Laboratories). The fluorescently stained samples were observed and photographed using an LSM-710 confocal microscope (Zeiss, Oberkochen, Germany).

#### 2.9.2. Immunohistochemistry

Embedded paraffin sections were deparaffinized, rehydrated, and incubated in serum solution for 1 h at room temperature to block nonspecific binding. The sections were then incubated overnight at 4 °C with primary antibodies (Table S1). The following day, after washing with PBS, the slides were incubated with biotinylated secondary antibodies (Vector Laboratories) for 1 h at room temperature, followed by additional washes with PBS. The ABC reagent (Vector Laboratories) was then applied, and the cells were washed again. Color development was performed using 3,3’-diaminobenzidine (Sigma-Aldrich) as the substrate, with a brown color indicating a positive reaction. Counterstaining was performed with hematoxylin (KPNT, Cheongju, Republic of Korea), and the slides were mounted using DPX mounting medium (Sigma-Aldrich). Finally, the slides were scanned, and images were acquired using a Motic Scan Infinity 100 system (Motic, Beijing, China).

#### 2.9.3. Hematoxylin and Eosin Staining

The paraffin-embedded tissue sections were deparaffinized, rehydrated, and stained with hematoxylin (KPNT) for 5 min. The sections were then rinsed, fractionated, and counterstained with eosin (KPNT) for 1 min. The stained sections were dehydrated, cleared, and mounted using DPX mounting medium (Sigma-Aldrich). Finally, the slides were scanned, and images were acquired using a Motic Scan Infinity 100 system (Motic).

The number of hair follicles and anagen follicle ratio was determined following the standardized morphological criteria [27]. The percentage of anagen follicles was calculated as the ratio of anagen follicles to the number of follicles in the measured fields.

### 2.10. Quantitative and Statistical Analyses

All image analyses were quantitatively analyzed using ImageJ software version 1.53s (NIH) and each group was compared with control. Data were expressed as mean ± standard deviation. Normality was assessed using the Shapiro–Wilk test. When the data did not follow a normal distribution, the Kruskal–Wallis test was used. The Mann–Whitney U test was used for comparisons between two groups. All statistical analyses were performed using SPSS v26 (IBM Corp., Armonk, NY, USA), and statistical significance is indicated in the legends of each figure.

## 3. Results

### 3.1. Chemical and Physicochemical Characterization of FYCE

The quantitative analysis revealed that the FYCE contained high levels of γ-aminobutyric acid (GABA; 50.9 ± 2.3 mg/g) and alanine (12.2 ± 0.5 mg/g), accounting for approximately 5% and 1% of the total free-amino-acid content, respectively (Table S1). A summary of FYCE characterization, including coliform and general bacterial analysis, and all parameters were within acceptable limits (Table S2).

Dynamic light scattering (DLS) measurements confirmed the presence of nanoscale colloidal populations within the FYCE, with intensity-based peaks detected around 100–200 nm. However, the Z-average hydrodynamic diameter was within the microscale range (~7 µm), and the very high PDI (1.0) indicated pronounced aggregation and substantial polydispersity, which limits the suitability of DLS alone for precise particle-size distribution analysis. Therefore, the microscale particle characteristics were further evaluated by laser diffraction, which revealed a predominant particle population with a mean diameter of 2.80 µm (d10 = 1.78 µm, d50 = 2.58 µm, d90 = 4.13 µm). Consistently, zeta potential analysis demonstrated a moderately negative surface charge (–12.7 mV), suggesting colloidal dispersion stability in aqueous medium (Figure S2).

FYCE is a heterogeneous multi-component colloidal complex with mixed nanoscale and microscale characteristics, consistent with fermented extracts containing peptides and polysaccharide-rich aggregates.

### 3.2. FYCE Increased NRF2 Expression and Decreased Oxidative Stress in H_2_O_2_-Treated DPCs

The optimal concentration of FYCE for in vitro experiments was determined based on its effect on DPC viability and NRF2 expression. Oxidative stress conditions were induced by treating DPCs with H_2_O_2_. 8-OHdG, a commonly used marker of oxidative stress [28], was significantly increased after treatment with 200 μM of H_2_O_2_ (Figure S3A,B).

This concentration was used to induce conditions of increased oxidative stress. The FYCE did not decrease cell viability up to 5 mg/mL in H_2_O_2_-treated DPCs. (Figure S3C,D).

Treatment with FYCE upregulated NRF2 expression in H_2_O_2_-treated DPCs. This effect was concentration dependent, reaching a maximum at 0.8 mg/mL, with no additional increase observed at higher concentrations. FYCE decreased 8-OHdG levels, showing the greatest decrease at 0.8 mg/mL in the H_2_O_2_-treated DPCs. Thus, 0.8 mg/mL FYCE was used in the subsequent experiments (Figure S3E–G).

**Figure 1:**
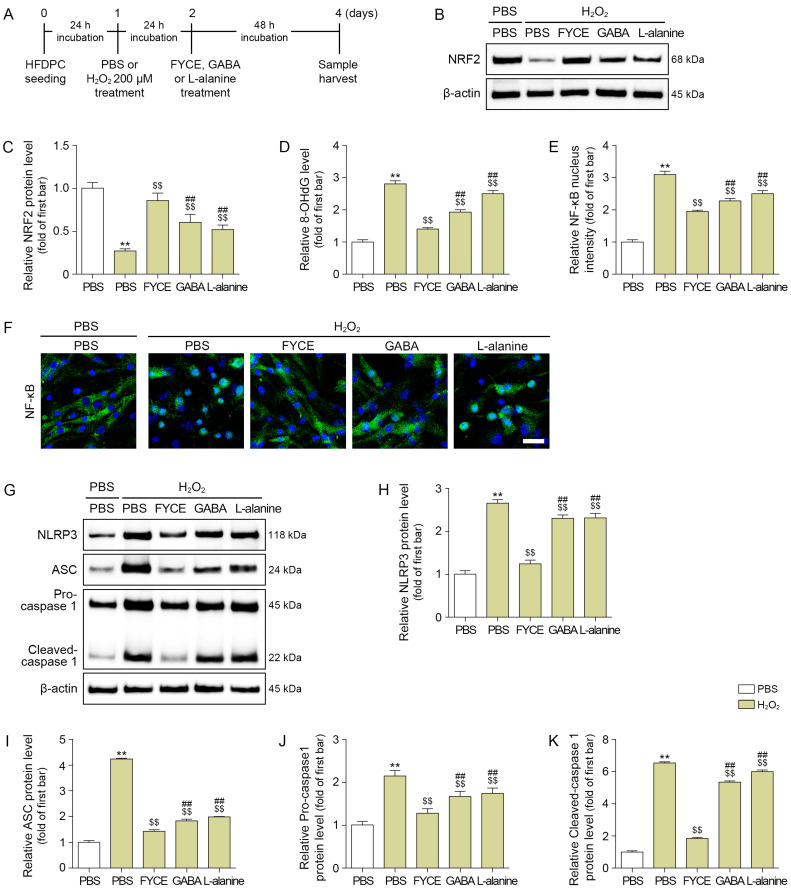
FYCE regulates oxidative stress, NF-kB activity, and NLRP3 inflammasome in H_2_O_2_-treated DPCs. (**A**) Schematic to determine the effects of FYCE, GABA, and L-alanine in H_2_O_2_-treated DPCs. (**B**,**C**) Western blot analysis of NRF2 after FYCE treatment in H_2_O_2_-treated DPCs. (**D**) ELISA analysis of 8-OHdG after FYCE treatment in H_2_O_2_-treated DPCs. (**E**,**F**) Immunocytochemistry analysis of NF-kB activity after FYCE treatment in H_2_O_2_-treated DPCs (Scale bar = 50 μm). (**G**–**K**) Western blot analysis of NLRP3 inflammasome after FYCE treatment in H_2_O_2_-treated DPCs. Data are expressed as the mean ± SD. **, *p* < 0.01, vs. first bar; $$, *p* < 0.01, vs. second bar; ##, *p* < 0.01, vs. third bar. 8-OHdG, 8-hydroxy-2’ -deoxyguanosine; ASC, apoptosis-associated speck-like protein containing a CARD; FYCE, fermented yeast complex extract; GABA, γ-aminobutyric acid; NF-κB, nuclear factor-kappa B; NLRP3, NOD-like receptor pyrin domain-containing protein 3; NRF2, nuclear factor erythroid 2-related factor 2; PBS, phosphate-buffered saline.

We treated H_2_O_2_-treated DPCs with GABA and L-alanine at the same concentrations as those found in the FYCE. Treatment with FYCE, GABA, or L-alanine increased NRF2 expression in H_2_O_2_-treated DPCs. The most prominent increase was observed with FYCE (Figure 1A–C).

H_2_O_2_ treatment increased 8-OHdG levels, which were decreased by treatment with FYCE, GABA, and L-alanine. The most prominent decrease was observed with FYCE (Figure 1D).

To determine whether the antioxidative effect of FYCE is distinct from that of YE, we additionally compared NRF2 expression in H_2_O_2_-treated DPCs following treatment with FYCE or YE administration. The NRF2 expression of FYCE was higher than that of YE (Figure S4).

### 3.3. FYCE Decreased NF-kB Activity and NLRP3 Inflammasome in H_2_O_2_-Treated DPCs

NF-κB activity was evaluated by measuring NF-κB intensity in the nuclei. NF-κB activity was increased by H_2_O_2_ treatment and decreased by FYCE, GABA, and L-alanine treatments. The most prominent effect was observed with FYCE (Figure 1E,F).

The NLRP3 inflammasome components (NLRP3, ASC, and Pro-caspase 1) and the level of the mature form of caspase 1 were increased by H_2_O_2_ treatment and decreased by treatment with FYCE, GABA, and L-alanine. The most prominent decrease was observed with FYCE (Figure 1G–K).

To validate the mechanistic role of NF-κB in oxidative-stress signaling, we first confirmed the efficacy of the NF-κB nuclear translocation inhibitor SN50 (Figure S5A). As shown in Figure S5B,C, H_2_O_2_ markedly increased nuclear NF-κB intensity, whereas SN50 substantially reduced NF-κB nuclear localization, demonstrating that the inhibitor functioned as expected. FYCE alone also suppressed NF-κB activation to a similar extent. Importantly, NF-κB levels in the SN50 + FYCE group did not differ statistically from those in the SN50-only group, indicating that NF-κB inhibition was already maximized by SN50 under these conditions (Figure S5A–C). Levels of 8-OHdG were further decreased in the SN50 + FYCE group compared with SN50 alone (Figure S5D), suggesting that FYCE exerts additional ROS-reducing effects beyond NF-κB inhibition. Consistent with this, NRF2 expression was highest in the SN50 + FYCE group, exceeding levels observed with SN50 alone (Figure S5E,F).

This enhancement of NRF2 activation under NF-κB inhibition suggests a potential positive feedback relationship between the two pathways, whereby the suppression of NF-κB facilitates NRF2 induction during oxidative stress.

### 3.4. FYCE Decreased Inflammatory Cytokines and Increased IGF-1 in H_2_O_2_-Treated DPCs

**Figure 2:**
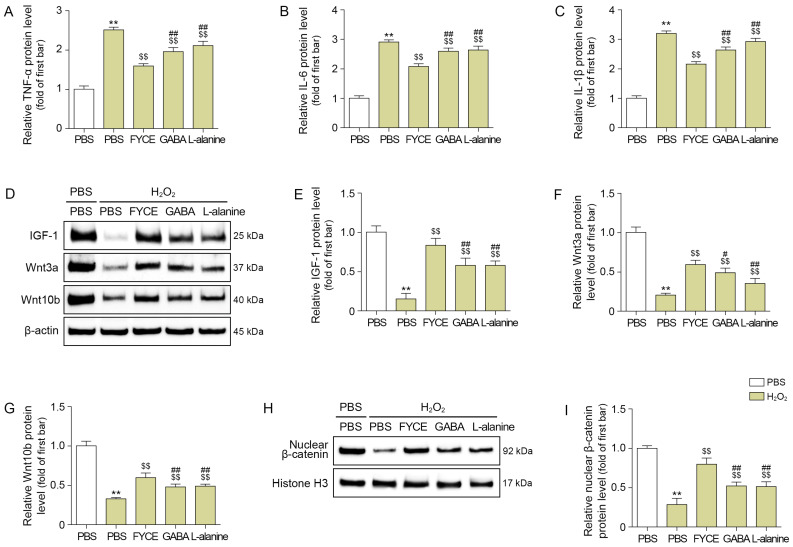
FYCE regulates inflammatory cytokines, IGF-1, and Wnt/β-catenin signaling in H_2_O_2_-treated DPCs. (**A**–**C**) ELISA analysis of TNF-α (**A**), IL-6 (**B**), and IL-1β (**C**) after FYCE treatment in H_2_O_2_-treated DPCs. (**D**–**G**) Western blot analysis of IGF-1, Wnt3a, and Wnt10b after FYCE treatment in H_2_O_2_-treated DPCs. (**H**,**I**) Western blot analysis of β-catenin after FYCE treatment in H_2_O_2_-treated DPCs. Data are expressed as the mean ± SD. **, *p* < 0.01, vs. first bar; $$, *p* < 0.01, vs. second bar; #, *p* < 0.05 and ##, *p* < 0.01, vs. third bar. FYCE, fermented yeast complex extract; GABA, γ-aminobutyric acid; IGF-1, insulin like growth factor 1; IL, interleukin; PBS, phosphate-buffered saline; TNF-α, tumor necrosis factor-α.

TNF-α, IL-6, and IL-1β were increased by H_2_O_2_ treatment and decreased by FYCE, GABA, and L-alanine treatments. The most prominent decrease was observed with FYCE (Figure 2A–C).

IGF-1 levels were decreased by H_2_O_2_ treatment and increased by treatment with FYCE, GABA, and L-alanine. The most prominent increase was observed with FYCE (Figure 2D,E).

The transition of hair follicles from the quiescent telogen phase to the active anagen phase is promoted by Wnt10b, a process mediated by the stabilization of β-catenin [29]. Similarly, Wnt3a stimulates β-catenin activity, resulting in accelerated hair growth [30].

The expression of Wnt3a and Wnt10b was decreased by H_2_O_2_ treatment and was increased by treatment with FYCE, GABA, and L-alanine (Figure 2D,F,G). The most prominent increasing effect was observed with FYCE. The expression of nuclear β-catenin was decreased by H_2_O_2_ treatment and increased with FYCE, GABA, and L-alanine treatments. The most prominent increase was observed with FYCE (Figure 2H,I).

### 3.5. FYCE Increased NRF2 and Decreased NF-kB Activity and NLRP3 Inflammasome in the Shaved Animal Skin

**Figure 3:**
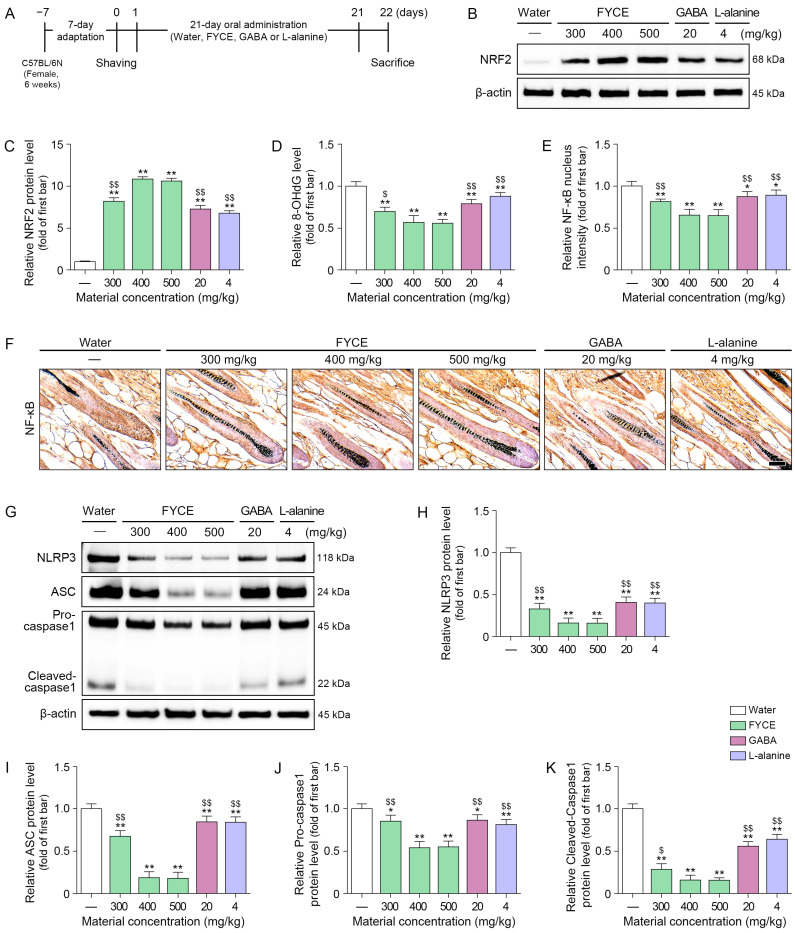
Oral administration of FYCE modulates NRF2, NF-κB activity, and NLRP3 inflammasome in animals. (**A**) Schematic to determine the effects of FYCE, GABA, and L-alanine in animals. (**B**,**C**) Western blot analysis of NRF2 after FYCE administration in animals. (**D**) ELISA analysis of 8-OHdG after FYCE administration in animals. (**E**,**F**) Immunocytochemistry analysis of NF-κB activity after FYCE administration in animals (Scale bar = 50 μm). (**G**–**K**) Western blot analysis of NLRP3 inflammasome after FYCE administration in animals. Data are expressed as the mean ± SD. *, *p* < 0.05 and **, *p* < 0.01, vs. first bar; $, *p* < 0.05 and $$, *p* < 0.01, vs. third bar. 8-OHdG, 8-hydroxy-2’ -deoxyguanosine; ASC, apoptosis-associated speck-like protein containing a CARD; FYCE, fermented yeast complex extract; GABA, γ-aminobutyric acid; NF-κB, nuclear factor-kappa B; NLRP3, NOD-like receptor pyrin domain-containing protein 3; NRF2, nuclear factor erythroid 2-related factor 2.

The administration of FYCE led to the upregulation of NRF2 expression in a dose-dependent manner at doses of up to 400 mg/kg. The NRF2 expression levels did not differ significantly between the 400 and 500 mg/kg treatment groups. For comparative analysis of the effects of FYCE, L-alanine, and GABA, we prepared a separate treatment group in which GABA (20 mg/kg) and L-alanine (4 mg/kg) were administered at concentrations equivalent to their proportions within the 400 mg/kg FYCE dose. The increasing NRF2 effect of all concentrations on FYCE was higher than that of GABA or L-alanine (Figure 3A–C).

8-OHdG levels were decreased by FYCE, GABA, and L-alanine treatments. These effects were most prominent with 400 and 500 mg/kg FYCE (Figure 3D).

NF-kB activity was decreased by FYCE, GABA, and L-alanine treatments. These effects were most prominent with 400 and 500 mg/kg FYCE (Figure 3E,F).

The levels of NLRP3 inflammasome components (NLRP3, ASC, and Pro-caspase 1) and Cleaved-caspase 1 were decreased by FYCE, GABA, and L-alanine treatments. These effects were most prominent with 400 and 500 mg/kg FYCE (Figure 3G–K).

### 3.6. FYCE Decreased Inflammatory Cytokines and Increased IGF-1 in the Shaved Animal Skin

**Figure 4:**
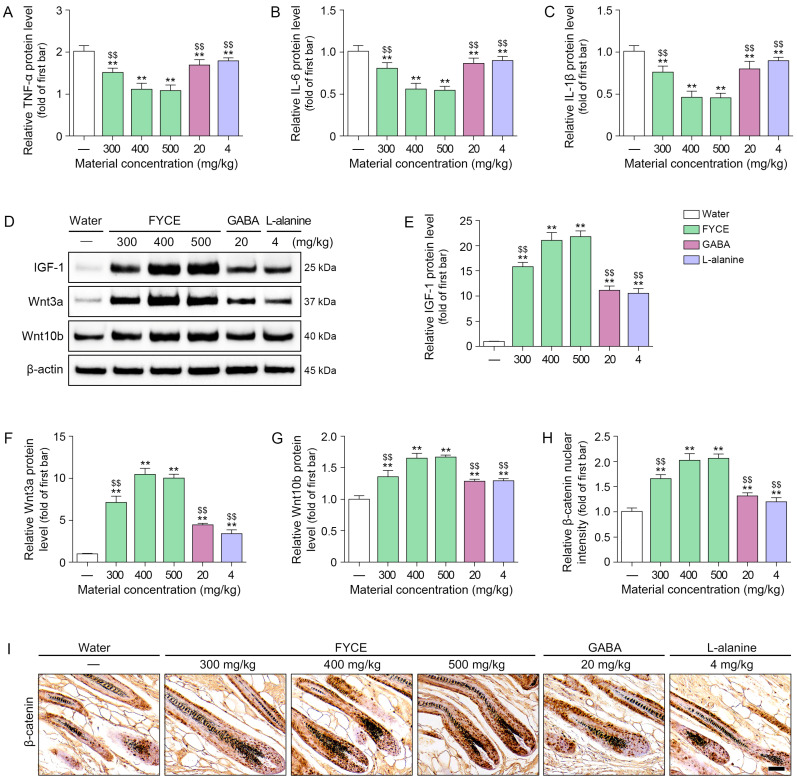
Oral administration of FYCE modulates inflammatory cytokines, IGF-1, and Wnt/β-catenin signaling in animals. (**A**–**C**) ELISA analysis of TNF-α (**A**), IL-6 (**B**), and IL-1β (**C**) after FYCE administration in animals. (**D**–**G**) Western blot analysis of IGF-1, Wnt3a, and Wnt10b after FYCE administration in animals. (**H**,**I**) Immunohistochemistry analysis of β-catenin after FYCE administration in animals (Scale bar = 50 μm). Data are expressed as the mean ± SD. **, *p* < 0.01, vs. first bar; $$, *p* < 0.01, vs. third bar. FYCE, fermented yeast complex extract; GABA, γ-aminobutyric acid; IGF-1, Insulin like growth factor 1; IL, Interleukin; TNF-α, tumor necrosis factor-α.

The levels of TNF-α, IL-6, and IL-1β were decreased by FYCE, GABA, and L-alanine. These effects were most prominent with 400 and 500 mg/kg FYCE (Figure 4A–C).

IGF-1, Wnt3a, Wnt10b, and β-catenin levels were increased by FYCE, GABA, and L-alanine treatment. These effects were most prominent with 400 and 500 mg/kg FYCE (Figure 4D–I).

### 3.7. FYCE Increased Hair Regrowth in the Shaved Animal Skin

**Figure 5:**
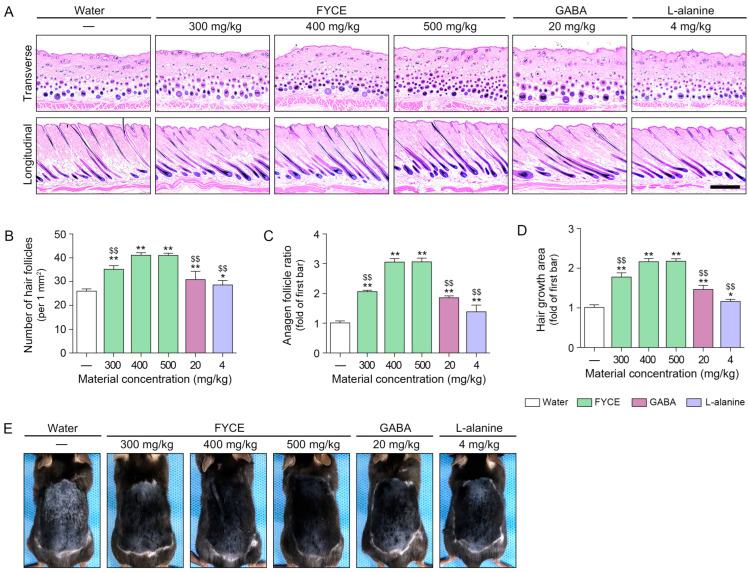
Oral administration of FYCE modulates hair follicle morphology and hair regrowth in animals. (**A**) Representative H&E staining results from transverse and longitudinal skin sections after FYCE administration in animals. Blue–purple indicates nuclei, and pink indicates cytoplasm (Scale bar = 500 μm). (**B**,**C**) Quantitative analysis of hair follicle number (**B**) and ratio of anagen follicle (**C**) in H&E-stained sections. (**D**,**E**) Representative images and quantitative analysis of dorsal hair regrowth in mice at the end of the experimental period. Data are expressed as the mean ± SD. *, *p* < 0.05 and **, *p* < 0.01, vs. first bar; $$, *p* < 0.01, vs. third bar. FYCE, fermented yeast complex extract; GABA, γ-aminobutyric acid.

The number of hair follicles was increased by FYCE, GABA, and L-alanine treatments. These effects were most prominent with 400 and 500 mg/kg FYCE (Figure 5A,B). FYCE, GABA, and L-alanine increased the ratio of follicles in the anagen phase (Figure 5A,C). These effects were most prominent with 400 and 500 mg/kg FYCE. The hair growth area was increased by FYCE, GABA, and L-alanine. These effects were most prominent with 400 and 500 mg/kg FYCE (Figure 5D,E).

To compare the hair-growth-promoting effect of FYCE with a well-established therapeutic agent, we evaluated FYCE alongside minoxidil, which served as the positive control. Western blot analysis demonstrated that NRF2 protein levels were lowest in the water group, elevated in mice treated with FYCE, and highest in the minoxidil-treated group (Figure S6A,B). FYCE increased the number of hair follicles compared with the water group, and minoxidil produced the greatest increase among the three groups (Figure S6C,D). Similarly, the proportion of anagen-phase follicles was higher in the FYCE group than in the water group and was further elevated in the minoxidil group (Figure S6E).

## 4. Discussion

Each hair follicle cycles independently, with an average of 10–30 cycles throughout an individual’s life [1]. A healthy scalp typically contains approximately 100,000 hairs at any given time, with a normal daily shedding rate of 100–150 telogen hairs [1]. As follicles are in various phases simultaneously, the overall hair density and count remain relatively stable [31]. The anagen phase is defined as the active production of a complete hair shaft from a follicle [32]. Consequently, hair length directly reflects the duration of this growth phase if the hair is not cut [32]. However, the duration of the anagen phase naturally decreases with age, leading to weaker and thinner hair over time [33]. This age-related change is also associated with a decline in the proportion of follicles in the anagen phase [34]. Notably, the premature termination of anagen growth, or anagen arrest due to an external insult, is considered an abnormal form of hair loss [31]. DP cells are essential for increasing HF and hair growth by modulating the hair follicle cycle [2]. Increased oxidative stress in DP leads to hair growth dysfunction; thus, various materials that decrease oxidative stress have been used to increase hair growth [35,36].

To evaluate whether FYCE, which contains the known antioxidants L-alanine and GABA, promotes hair regrowth by reducing oxidative stress, we induced oxidative stress in DPC using H_2_O_2_. We confirmed that FYCE treatment increased NRF2 expression and decreased oxidative stress, as measured by 8-OHdG levels. FYCE also reduced NF-κB activity and the expression of its downstream targets, TNF-α and IL-6. Furthermore, FYCE suppressed the formation of the NLRP3 inflammasome and consequently reduced the level of IL-1β.

Reports have suggested that the NLRP3 inflammasome influences hair follicles and contributes to hair loss [37,38]. These findings are primarily based on experiments using animal models of alopecia, particularly those caused by inflammation [37,38]. Alopecia areata is an autoimmune non-scarring hair loss disease, and an alopecia areata animal model can be generated using C3H/HeJ mice by adding inflammatory aggravation factors such as chronic stress or the injection of lymph node cells [37,38]. In alopecia areata animals, the NLRP3 inflammasome increased, and the NLRP3 inflammasome inhibitor (MCC950) decreased hair loss [37].

Previous studies have shown that NLRP3 inflammasome activation is increased during hair loss caused by inflammation, which was induced by the treatment of hair follicles with strong inflammatory agents. In contrast, our study observed that the NLRP3 inflammasome was also formed by oxidative stress induced by H_2_O_2_ without the use of inflammation-inducing agents, leading to an increase in various inflammatory cytokines.

Oxidative stress activates the NF-κB signaling pathway, which subsequently increases inflammation [17]. Therefore, NF-κB can be considered a central mediator linking oxidative stress and inflammatory responses. In line with this, accumulating evidence indicates that NRF2 and NF-κB form a bidirectional regulatory network under oxidative stress. The activation of NRF2 enhances the expression of antioxidant and cytoprotective enzymes, lowers intracellular ROS, and thereby limits NF-κB activation, in part by preventing IκBα degradation [39]. Conversely, the NF-κB p65 subunit can directly repress the NRF2–ARE pathway at the transcriptional level by competing with NRF2 for the co-activator CBP and by recruiting HDAC3 to NRF2 target promoters, leading to reduced NRF2-dependent gene expression [40]. Thus, excessive NF-κB activity not only amplifies inflammation, but can also dampen NRF2-mediated antioxidant responses.

Our inhibitor experiment with the NF-κB nuclear translocation blocker SN50 supports this reciprocal crosstalk. SN50 effectively reduced NF-κB nuclear localization in H_2_O_2_-treated DPCs, suggesting that the pathway is functionally engaged. Although co-treatment with FYCE did not further decrease NF-κB nuclear intensity beyond SN50 alone, the combination produced a greater reduction in the oxidative DNA damage marker 8-OHdG and yielded the highest NRF2 protein levels among all H_2_O_2_-treated groups. These findings suggest that FYCE not only attenuates NF-κB activation indirectly via NRF2-driven ROS reduction, but may also exploit the relief of NF-κB–mediated repression to further enhance NRF2 signaling.

Moreover, NF-κB plays a crucial role in the priming step of NLRP3 inflammasome activation by acting as a transcription factor [41]. Following an inflammatory stimulus, NF-κB translocates to the nucleus and upregulates the expression of key inflammasome components, including the NLRP3 protein and the precursor form of the cytokine pro-IL-1β [41].

In this study, FYCE increased NRF2, decreased oxidative stress, and decreased NF-κB activity and NLRP3 inflammasome formation in shaved animal skin. Moreover, TNF-α, IL-6, and IL-1β were decreased in the shaved animal skin. In addition to decreasing inflammation, IGF-1 expression was increased by FYCE in both H_2_O_2_-treated DPCs and shaved animal skin.

IGF-1 stabilizes β-catenin by activating the PI3K/Akt signaling pathway, which leads to the phosphorylation and inactivation of GSK-3β [42]. This inactivation prevents the “destruction complex” from tagging β-catenin for proteasomal degradation [43]. Consequently, stabilized β-catenin accumulates in the cytoplasm and translocates to the nucleus to act as a co-transcriptional factor with LEF/TCF, promoting the expression of genes essential for cell proliferation and hair follicle anagen induction [44].

Although FYCE increased anagen-related factors such as Wnt3a and Wnt10b in both H_2_O_2_-treated DPCs and shaved animal skin, and enhanced hair regrowth by increasing the number of anagen-phase follicles, its efficacy was comparatively weaker than that of minoxidil. In our comparative experiment, FYCE did not elevate NRF2 expression to the same extent as minoxidil, nor did it induce a greater increase in total follicle number or anagen-phase follicles. However, despite its strong hair-regrowth efficacy, minoxidil is known to be associated with several practical limitations, including scalp irritation, contact dermatitis, dryness, and cosmetic inconvenience due to daily topical application [45,46]. Systemic side effects such as headaches, hypertrichosis in unintended areas, and occasional cardiovascular effects have also been reported [47]. These drawbacks can limit long-term adherence and reduce overall patient satisfaction. Given these limitations, a milder agent such as FYCE—although less potent than minoxidil—may still be valuable as a complementary or alternative option.

Evidence suggests that oxidative stress is a significant factor in hair loss pathogenesis [48]. Consequently, antioxidant supplementation, administered either topically or orally, has been widely investigated as a therapeutic strategy to mitigate hair loss [49]. Salvianolic acid B, a polyphenolic compound derived from *Salvia miltiorrhiza*, was recently shown to promote hair growth by reducing ROS levels and activating NRF2 signaling in dermal papilla cells and in murine models [35]. Niacinamide has also been reported to protect DPCs from oxidative injury by downregulating DKK-1 and attenuating ROS-induced NF-κB activation [36].

A clinical trial evaluating an oral nutritional supplement, a combination of specific omega-3 and omega-6 fatty acids from fish and blackcurrant seed oils along with lycopene, vitamin C, and vitamin E, demonstrated a positive effect on hair health [50]. This treatment significantly increased hair density by reducing the number of telogen follicles and miniaturizing hair follicles [50]. Topical applications have also been explored, with a new active blend lotion containing two polyphenol components, dihydroquercetin glucoside and epigallocatechin gallate glucoside, showing promise for the treatment of androgenetic alopecia [51].

Topical hair growth formulations have certain limitations, including poor skin penetration and potential for decreased absorption [52]. Depending on the formulation, they can leave behind a sticky residue on the scalp or interfere with hair styling. Therefore, oral supplements may offer a higher degree of patient compliance and less inconvenience than topical products.

Dietary supplements and functional foods are generally considered safer than pharmaceuticals [53]. FYCE is produced through the fermentation of brewer’s yeast, kelp extract, and fish collagen [54,55]. Although the antioxidant and anti-inflammatory effects of yeast extracts have been reported before [56,57], FYCE demonstrates distinct biochemical and functional characteristics resulting from its dual-fermentation system. The sequential fermentation with *L. brevis* BJ20 and *L. plantarum* BJ21 facilitates the conversion of glutamate to GABA and the hydrolysis of collagen into short bioactive peptides that enhance cellular antioxidant defense via NRF2 activation and suppress NF-κB signaling. In addition, the incorporation of kelp extract provides iodine and other marine-derived micronutrients that further support redox balance and anti-inflammatory responses. Consistent with these compositional advantages, our comparative experiments using YE showed that FYCE induced a greater increase in NRF2 expression, confirming that FYCE exerts superior antioxidative activity relative to conventional YE. Collectively, these features define FYCE as a novel multi-component fermented complex with superior bioactivity compared with conventional YE, thereby confirming its originality and potential as a functional ingredient for hair-growth applications.

Importantly, FYCE’s physicochemical properties may contribute to its biological activity. The formulation exhibited a bimodal size distribution, consisting of nanoscale components (~108 nm) and stable microscale aggregates (mean 2.80 µm; d10–d90: 1.78–4.13 µm), along with a moderately negative zeta potential (–12.7 mV). Such heterogeneous colloidal architecture is typical of fermented multi-component complexes and can influence dispersibility and interaction with biological barriers. Nano-sized fractions have been reported to facilitate closer cellular interaction and improved intestinal uptake after oral administration [58,59], whereas micro-sized particles may provide prolonged retention or sustained release at target tissue sites [60].

Both brewer’s yeast and fish collagen have a long history of use as oral supplements and are known to have low toxicity. Fermented kelp extract is also widely used as an oral supplement, but due to its iodine content, cautious consumption is recommended for pregnant and lactating women [61].

Although the individual components of FYCE, brewer’s yeast, kelp extract, and fish collagen, are known to have low toxicity and have long been used as oral supplements, the unique combination and fermentation process could potentially alter the safety profile of FYCE. As the primary goal of this study was to elucidate the mechanism by which FYCE reduces oxidative stress to promote hair growth and identify the mediating cellular signals, we did not perform in vivo toxicity studies. Further studies on FYCE toxicity are essential to confirm its safety in hair regrowth applications.

Another limitation of this study is that the in vivo evaluation was only performed under physiological anagen induction in shaved mice, without applying an exogenous oxidative or inflammatory insult. Although the FYCE modulated NRF2, NF-κB, and NLRP3 signaling in both H_2_O_2_-treated DPCs and shaved skin, future studies utilizing established oxidative- or inflammation-induced hair-loss models will be necessary to determine whether FYCE can rescue or reverse experimentally induced follicular damage.

Despite demonstrating that FYCE reduces oxidative stress and inflammation through NRF2 activation and the suppression of NF-κB/NLRP3 signaling, the precise upstream molecular mechanisms remain incompletely understood. In particular, the receptor-level interactions and intracellular intermediates modulated by GABA and L-alanine in dermal papilla cells have not been fully elucidated. Further mechanistic studies will be necessary to define how each component of FYCE contributes to its overall bioactivity.

Despite these limitations, our study established that FYCE promoted hair regrowth by increasing NRF2 expression and reducing oxidative stress. This, in turn, decreased NF-κB activity and NLRP3 inflammasome formation, both of which can be upregulated by oxidative stress. Furthermore, FYCE treatment increased IGF-1 expression. We speculate that the GABA and L-alanine contained in FYCE are the primary contributing factors to these effects. When administered alone, GABA and L-alanine promote hair regrowth, albeit to a lesser extent than FYCE. The superior efficacy of FYCE compared with that of its individual components suggests a synergistic effect between its constituents. Our analysis using the Bliss Independence Model showed a synergistic effect between GABA and L-alanine. However, it cannot be definitively concluded that this synergy solely accounts for the superior hair growth-promoting effect of FYCE compared to individual GABA or L-alanine treatments, as FYCE contains other components that were not investigated in this study. Therefore, the unexamined constituents of FYCE may also contribute to its enhanced efficacy in stimulating hair regrowth.

## 5. Conclusions

In conclusion, in this study, we demonstrated that FYCE effectively mitigates oxidative stress and promotes the activation of key hair growth signaling pathways in DPCs, which are essential for hair follicle regeneration. These results highlight the potential value of FYCE as a novel agent for the prevention and treatment of hair loss. Future investigations, including in vivo and clinical studies, are warranted to confirm its therapeutic utility and explore its application in more complex biological systems. Yeast-derived bioactive materials have been previously investigated for diverse applications, including antioxidant support, immune modulation, and skin barrier enhancement, suggesting that FYCE may also hold broader utility beyond hair biology. Future studies exploring these additional applications may further expand the relevance of FYCE as a multifunctional fermented complex.

## Data Availability

All data are contained within the article.

## Supplementary Materials

The following supporting information can be downloaded at https://www.mdpi.com/article/10.3390/antiox14121503/s1. Table S1. Free amino acid composition of FYCE (Top 5 components); Table S2. Quality evaluation of FYCE; Table S3. List of antibodies for enzyme-linked immunosorbent assay (ELISA), western blot (WB) and immunocytochemistry (ICC)/immunohistochemistry (IHC); Figure S1. FYCE manufacturing workflow; Figure S2. Physicochemical characterization of FYCE; Figure S3. Confirmation of oxidative stress and effective concentration of FYCE in DPCs; Figure S4. Comparative effects of FYCE and YE on NRF2 expression in H_2_O_2_-treated DPCs; Figure S5. FYCE regulates NF-kB activity and oxidative stress NF-κB inhibition under in H_2_O_2_-treated DPCs; Figure S6. Comparative effects of FYCE and minoxidil on NRF2 expression and hair regrowth in animals.
