# Supplementary material for: Fermented Yeast Complex Extract Promotes Hair Regrowth by Decreasing Oxidative Stress
Source: Antioxidants (Basel). 2025 Dec 14;14(12):1503. doi: 10.3390/antiox14121503 (PMC12729646; doi:10.3390/antiox14121503)
Supplement: Supplementary file 1 [file antioxidants-14-01503-s001.zip › antioxidants-3957515-supplementary.pdf]

Table S1. Free amino acid composition of FYCE (Top 5 components).

Reagent	mg/g	%
γ-amino-n-butyric acid	50.9	5.1
Alanine	12.2	1.2
Leucine	6.88	0.7
Valine	5.54	0.5
Phosphoethanolamine	4.72	0.4

The free amino acid profile of the FYCE was analyzed, and the five most abundant amino acid were identified. Among the detected amino acid, γ-amino-n-butyric acid, Alanine, Leucine, Valine and phosphoethanolamine were predominant. FYCE, Fermented yeast complex extract.

Table S2. Quality evaluation of FYCE.

Item	Specification	Result	Judgement	Remark
Appearance	Characteristic odor and uniform brown color, free form foreign matter	Characteristic odor and uniform brown color, free form foreign matter	Acceptable	–
Foreign matter	Not detected	Not detected	Acceptable	Confirmed in stability test
Total coliforms	Negative	Negative	Acceptable	–

The formulation exhibited a characteristic odor and uniform brown color without any foreign matter, consistent with the specified standard. No foreign substances were detected, and the total coliform test was negative, confirming microbiological safety. All parameters met the acceptance criteria and were therefore judged as compliant. FYCE, Fermented yeast complex extract.

Table S3. List of antibodies for enzyme-linked immunosorbent assay (ELISA), western blot (WB) and immunocytochemistry (ICC) / immunohistochemistry (IHC).

Antibody	Company	Dilution rate		
		ELISA	WB	ICC/IHC
β-actin	Cell signaling	-	1:1,000	-
NRF2	Bioss		1:1,000	-
8-OHdG	Abcam		1:500	-
NF-κB	Cell signaling	-	-	1:200
NLRP3	Boster	-	1:1,000	-
ASC	Santa cruz	-	1:1,000	-
Caspase 1	Santa cruz	-	1:1,000	-
TNF-α	Novusbio	1:500	-	-
IL-6	Fine Test	1:500	-	-
IL-1β	Bioss	1:500	-	-
IGF-1	Abcam	-	1:500	-
Wnt3a	Bioss	-	1:1,000	-
Wnt10b	Bioss	-	1:1,000	-
β-catenin	Santa cruz	-	1:500	1:50
Histone H3	Abcam	-	1:1,000	-

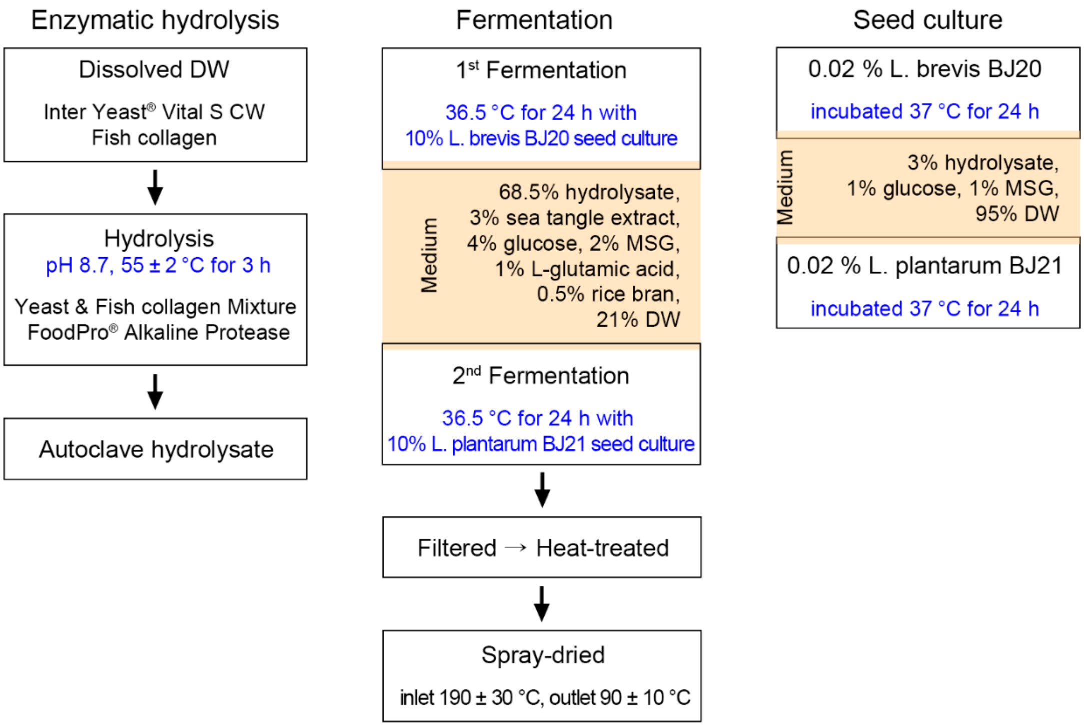

Figure S1. FYCE manufacturing workflow. Hydrolysate production, seed culture preparation, and two-stage Fermentation. DW, distilled water; FYCE, fermented yeast complex extract; MSG, monosodium glutamate.

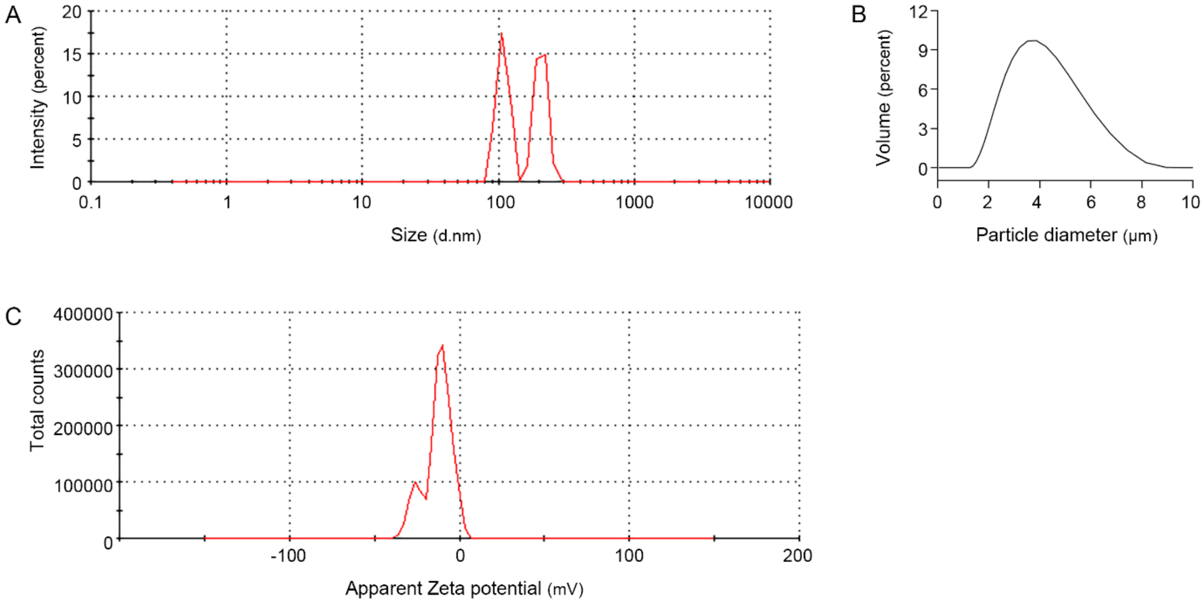

Figure S2. Physicochemical characterization of FYCE. (**A**) DLS intensity-weighted size distribution of FYCE, showing the presence of a nanoscale colloidal population. (**B**) Laser diffraction particle-size distribution analysis of FYCE, indicating a predominant micro-sized particle population (d10 = 1.78 μm, d50 = 2.58 μm, d90 = 4.13 μm). (**C**) Zeta potential distribution of FYCE, demonstrating a moderately negative surface charge (mean −12.7 mV) in aqueous suspension. DLS, dynamic light scattering; FYCE, fermented yeast complex extract.

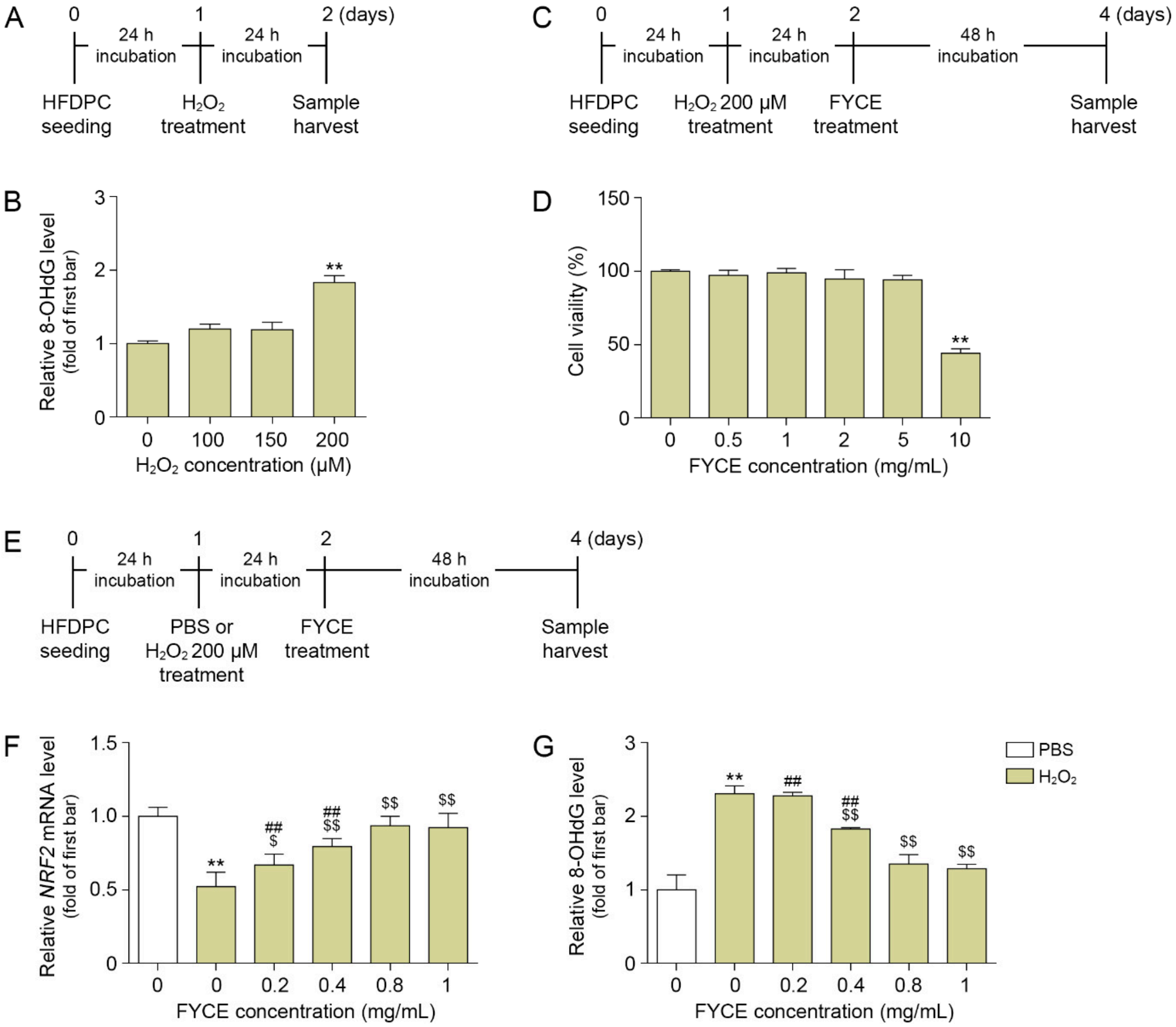

Figure S3. Confirmation of oxidative stress and effective concentration of FYCE in DPCs. (**A**) Schematic for confirming oxidative stress in HFDPC. (**B**) Determination of the H_2O_2 concentration that induces oxidative stress by analyzing 8-OHdG levels in DPCs using ELISA. (**C**) Schematic for confirming the cytotoxicity of FYCE in DPCs. (**D**) Confirmation of cytotoxicity at various concentrations of FYCE. (**E**) Schematic for confirming the effective concentration of FYCE in HFDPC. (**F**) Changes in NRF2 RNA expression following FYCE treatment. (G) Changes in 8-OHdG levels following FYCE treatment. Data are expressed as the mean ± SD. **, $p < 0.01$, vs. first bar; $, $p < 0.05$ and $$, $p < 0.01$, vs. second bar; ##, $p < 0.01$, vs. sixth bar. FYCE, fermented yeast complex extract; NRF2, nuclear factor erythroid 2-related factor 2; PBS, phosphate-buffered saline.

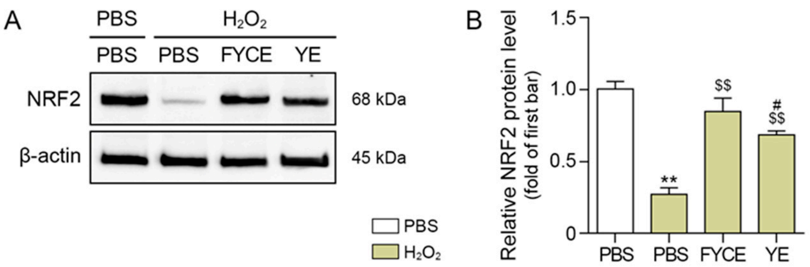

Figure S4. Comparative effects of FYCE and YE on NRF2 expression in H_2O_2-treated DPCs. (**A,B**) Western blot analysis of NRF2 after FYCE or YE treatment in H_2O_2-treated DPCs. Data are expressed as the mean ± SD. **, $p < 0.01$, vs. first bar; $$, $p < 0.01$, vs. second bar; #, $p < 0.05$, vs. third bar. FYCE, fermented yeast complex extract; NRF2, nuclear factor erythroid 2-related factor 2; PBS, phosphate-buffered saline.

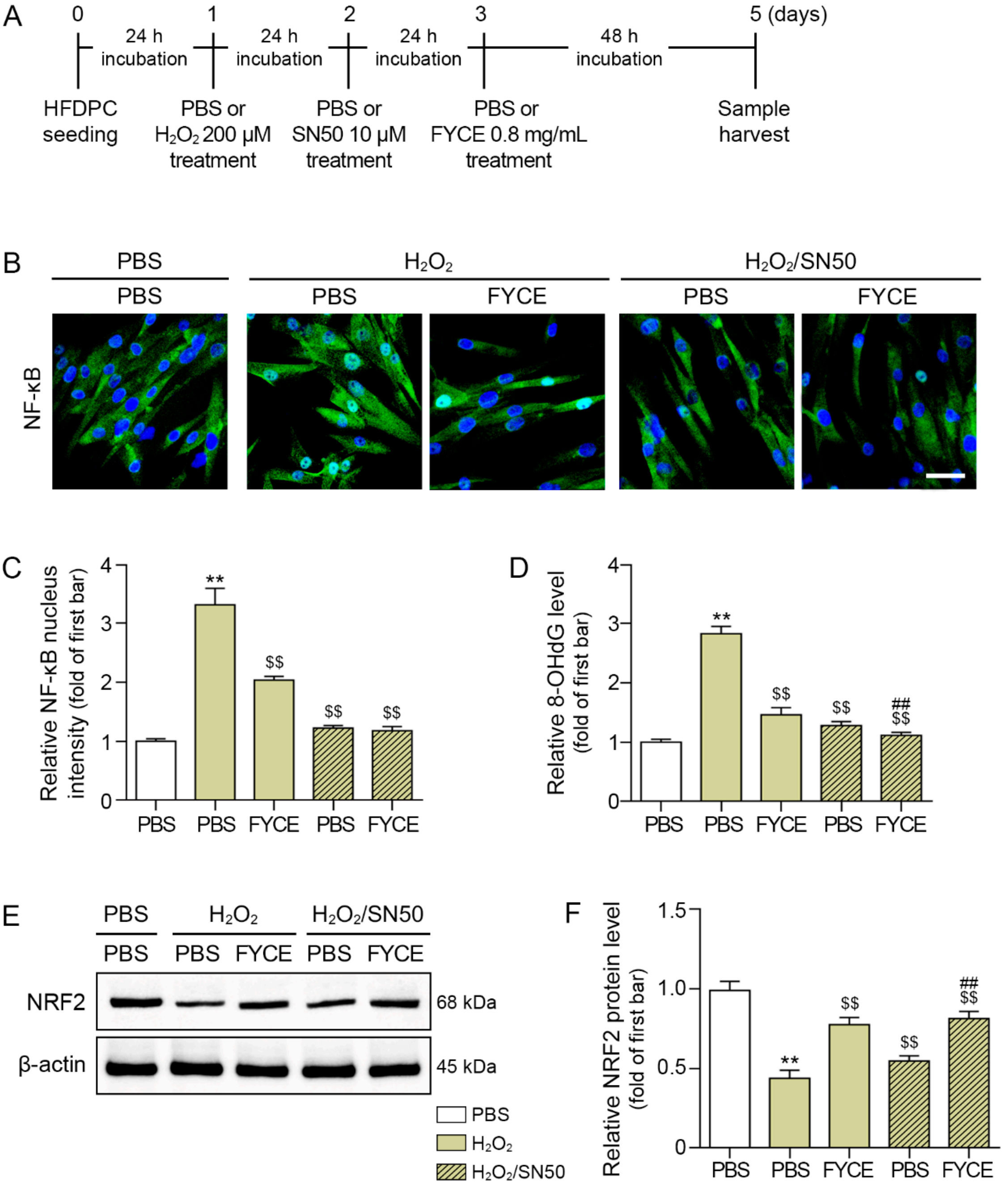

Figure S5. FYCE regulates NF-kB activity and oxidative stress NF-κB inhibition under in H_2O_2-treated DPCs. (**A**) Schematic to determine the effects of SN50 and FYCE in H_2O_2-treated HFDPC. (**B**,**C**) Immunocytochemistry analysis of NF-kB activity (scale bar = 50 μm). (**D**) ELISA analysis of 8-OHdG after FYCE treatment. (**E**,**F**) Western blot analysis of NRF2 after FYCE treatment. Data are expressed as the mean ± SD. **, $p < 0.01$, vs. first bar; $$, $p < 0.01$, vs. second bar; ##, $p < 0.01$, vs. fourth bar. 8-OHdG, 8-hydroxy-2' -deoxyguanosine; FYCE, fermented yeast complex extract; NF-κB, nuclear factor-kappaB; NRF2, nuclear factor erythroid 2-related factor 2; PBS, phosphate-buffered saline.

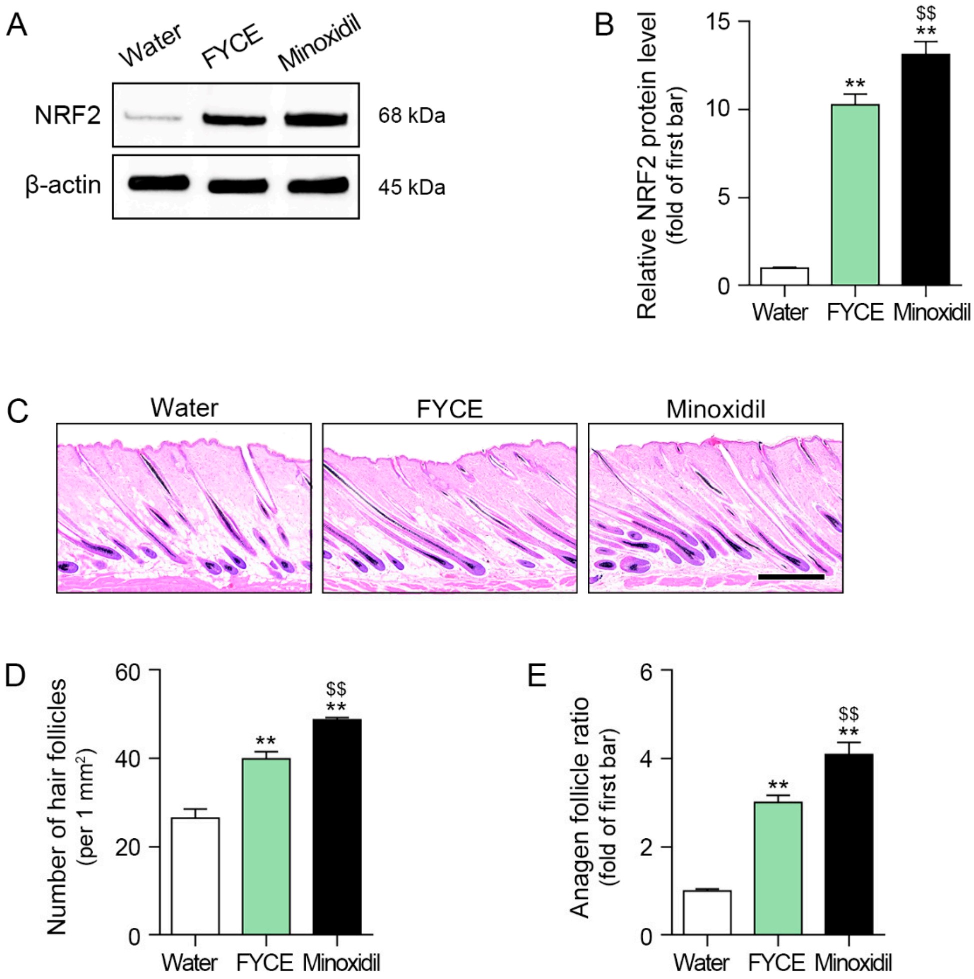

Figure S6. Comparative effects of FYCE and minoxidil on NRF2 expression and hair regrowth in animals. (**A**,**B**) Western blot analysis of NRF2 in animals. (**C**–**E**) Quantitative analysis of hair follicle number (**D**) and ratio of anagen follicle (**E**) in H&E-stained sections (**C**). Data are expressed as the mean ± SD. **, $p < 0.01$, vs. first bar; $$, $p < 0.01$, vs. second bar. FYCE, fermented yeast complex extract; GABA, γ-aminobutyric acid.
